# Clinical recovery is not a requirement for subjective well-being: a longitudinal study in older Dutch patients living with schizophrenia

**DOI:** 10.1192/j.eurpsy.2022.323

**Published:** 2022-09-01

**Authors:** P.D. Meesters, D. Rhebergen

**Affiliations:** 1 GGZ Friesland, Van Andel Ouderenpsychiatrie, Leeuwarden, Netherlands; 2 GGZ Centraal, Education, Amersfoort, Netherlands

**Keywords:** clinical recovery, Older Adults, Subjective Well-Being, schizophrénia

## Abstract

**Introduction:**

Growing old with schizophrenia is a profound challenge. However, impact and course of the disorder vary widely among individuals. The recovery concept has inspired outcome evaluation. In older schizophrenia patients, research on recovery is limited and mainly cross-sectional.

**Objectives:**

To compare 5-year outcome of clinical recovery (symptomatic remission and adequate community functioning) with outcome of subjective well-being (as a key element of personal recovery).

**Methods:**

Our catchment-area based study sample consisted of 73 older Dutch schizophrenia patients (mean age 65.9 years; SD 5.4), including both community living and institutionalized patients regardless of the age of onset of their disorder.

**Results:**

At baseline (T1) 5.5% of participants qualified for clinical recovery, while at five-year follow-up (T2) this rate was 12.3% (p=0.18, exact McNemar’s test). Subjective well-being was reported by 20.5% of participants at T1, and by 27.4% at T2 (p=0.27, exact McNemar’s test). Concurrent clinical recovery and subjective well-being was exceptional, being present in only one participant at T1 and in two participants at T2. Clinical recovery and subjective well-being were not correlated at T1 (p=0.82; phi=0.027), nor at T2 (p=0.71; phi= -0.044).

**Conclusions:**

Transitions over time confirm a dynamic course of schizophrenia in later life, with room for improvement. In our sample, we found no linkage between clinical recovery and subjective well-being. Results suggest that while clinical recovery is relatively rare in older individuals with schizophrenia it is not a prerequisite to experience subjective well-being. In spite of ongoing symptoms a substantial number of older schizophrenia patients report subjective well-being and thus may find ‘wellness within illness’.

**Disclosure:**

No significant relationships.

